# Mosaic chromosome 18 anomaly delineated in a child with dysmorphism using a three-pronged cytogenetic techniques approach: a case report

**DOI:** 10.1186/s12920-020-00796-9

**Published:** 2020-09-24

**Authors:** Harsh Sheth, Sunil Trivedi, Thomas Liehr, Ketan Patel, Deepika Jain, Jayesh Sheth, Frenny Sheth

**Affiliations:** 1 FRIGE’s Institute of Human Genetics, FRIGE House, Satellite, Ahmedabad, 380015 India; 2 University Clinic Jena, Institute of Human Genetics, Am Klinikum 1, 07747 Jena, Germany; 3 Himalaya Arcade A, Vastrapur, Ahmedabad, 380015 India; 4 Shishu Child Development & Early Intervention Centre, 403, Addor Ambition, Navrangpura, Ahmedabad, 380014 India

**Keywords:** Molecular karyotyping, Microarray, Molecular cytogenetics, Multi-color banding, Ring chromosome r(18), Mosaic chromosome 18, Case report

## Abstract

**Background:**

A plethora of cases are reported in the literature with iso- and ring-chromosome 18. However, co-occurrence of these two abnormalities in an individual along with a third cell line and absence of numerical anomaly is extremely rare.

**Case presentation:**

A 7-year-old female was referred for diagnosis due to gross facial dysmorphism and severe developmental delay. She presented with dysmorphic features, hypo/hyper pigmentation of the skin, intellectual disability and craniosynostosis. G-banding chromosome analysis suggested mos 46,XX,psu idic(18)(p11.2)[25]/46,XX,r(?18)[30]. Additional analysis by molecular karyotyping suggested pure partial deletion of 15 Mb on 18p (18p11.32p11.21). Lastly, multiple rearrangements and detection of a third cell line (ring chr18 and interstitial deletion) of chr18 was observed by multi-color banding.

**Conclusion:**

The current study presents a novel case of chromosomal abnormalities pertaining to chromosome 18 across 3 cell lines, which were delineated with a combinatorial approach of diagnostic methods.

## Background

Structural anomalies involving chromosome 18 (chr18) are relatively frequently observed with an incidence at birth of ~ 1/40,000 [1–4]. These anomalies are grouped into 18q-, 18p-, ring 18 and tetrasomy 18p [5]. Ring chromosome 18 (r18) has been reported in several cases where a normal maternal or paternal chromosome is replaced by r18. Majority of the patients are female and present with a milder clinical phenotype compared to patients with chromosome 18 long-arm deletions [6]. In most cases, the inherent instability of ring chromosomes leads to loss of the ring, double ring, and/or multiple copies of the ring chromosome in a variable percentage of cells [7]. r18 and isochromosome anomaly individually are well documented in the literature. However, their occurrence together as mosaic cell lines have been reported only in a few cases, yet [8–10]. Isochromosome 18q (92% cells) along with r18 (8% cells) was reported by Souraty et al. [8] in a girl with congenital anomalies. Madan et al. [9] reported a baby girl with mosaicism consisting of cells of an iso-pseudo dicentric chromosome 18 (psu idic(18)(p11). Another case (a baby girl) with triple mosaicism involving chromosome 18 [46,XX,del (18)(p11.23)/46,XX,idic(18)(p11.23)/46,XX,r (18)] was reported by Bocian et al. [10]. Similarly, co-occurrence of r18 and an acentric fragment are also detected rarely [11]. It is rare to observe multiple structural rearrangements replacing chr18 in the absence of numerical alterations. We report here a novel case with gross dysmorphism and developmental delay in which we observed a mosaic karyotype consisting of cells with an psu idic(18)(p11.2) together with a r18 and a derivative chr18 with interstitial deletion replacing the respective chromosome 18 in a mosaic pattern.

## Case presentation

**Fig. 1:**
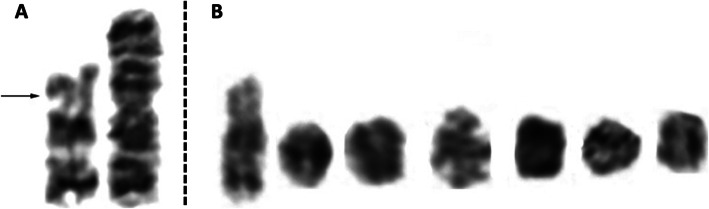
Partial G-banded karyotype showing two cell lines as mos 46,XX,iso(18)(p11.2)[25]/46,XX,r(?18)[30]dn. **a** Pseudo isodicentric chr18 and (**b**) ring chromosome of various sizes

A female child was referred for diagnosis at the age of 7 years due to dysmorphism and severe developmental delay. She was the first child born to a non-consanguineous young couple [father age: 28 years, mother age 26 years at birth of the proband] with an uneventful natural pregnancy. She was born full term but with delayed crying i.e. breathing [APGAR: n.a.] and a birth weight of 2.5 kg. Her development timeline showed her ability to stand at 8 months, walk unaided at 2.5 years and speak only bi-syllable words at the time of referral. Her height was 103 cm, weight of 15 kg and head circumference of 49 cm. She presented with several dysmorphic features, most notably: mild asymmetry of face, squint eyes, large ears, depressed nasal bridge, flat occiput, as well as webbed neck, broad trunk, wide nipple, pectus excavatum, scoliosis and hypo/hyper pigmentation on skin. Intellectual disability was also observed as she was unable to understand the verbal commands as per her age. Craniosynostosis was detected on the MRI. Peripheral blood sample collection and written informed consent was obtained in accordance with the Helsinki declaration. Chromosome analysis of the patient and parents was performed with 72 h lymphocyte culture and standard GTG-banding, which revealed a mosaicism involving two cell lines. The karyotype was interpreted according to the International System for Human Cytogenomic Nomenclature (ISCN 2016) [12] as mos 46,XX,psu idic(18)(p11.2)[25]/46,XX,r(?18)[30]. The presence of pseudoisodicentric chr18 was confirmed by both C-banding and fluorescence in situ hybridization (FISH) using a centromere 18 specific probe (data not shown). Karyotype of the parents was normal, confirming a de novo origin of the rearranged chromosome 18 (Fig. 1 a and b).

**Fig. 2:**
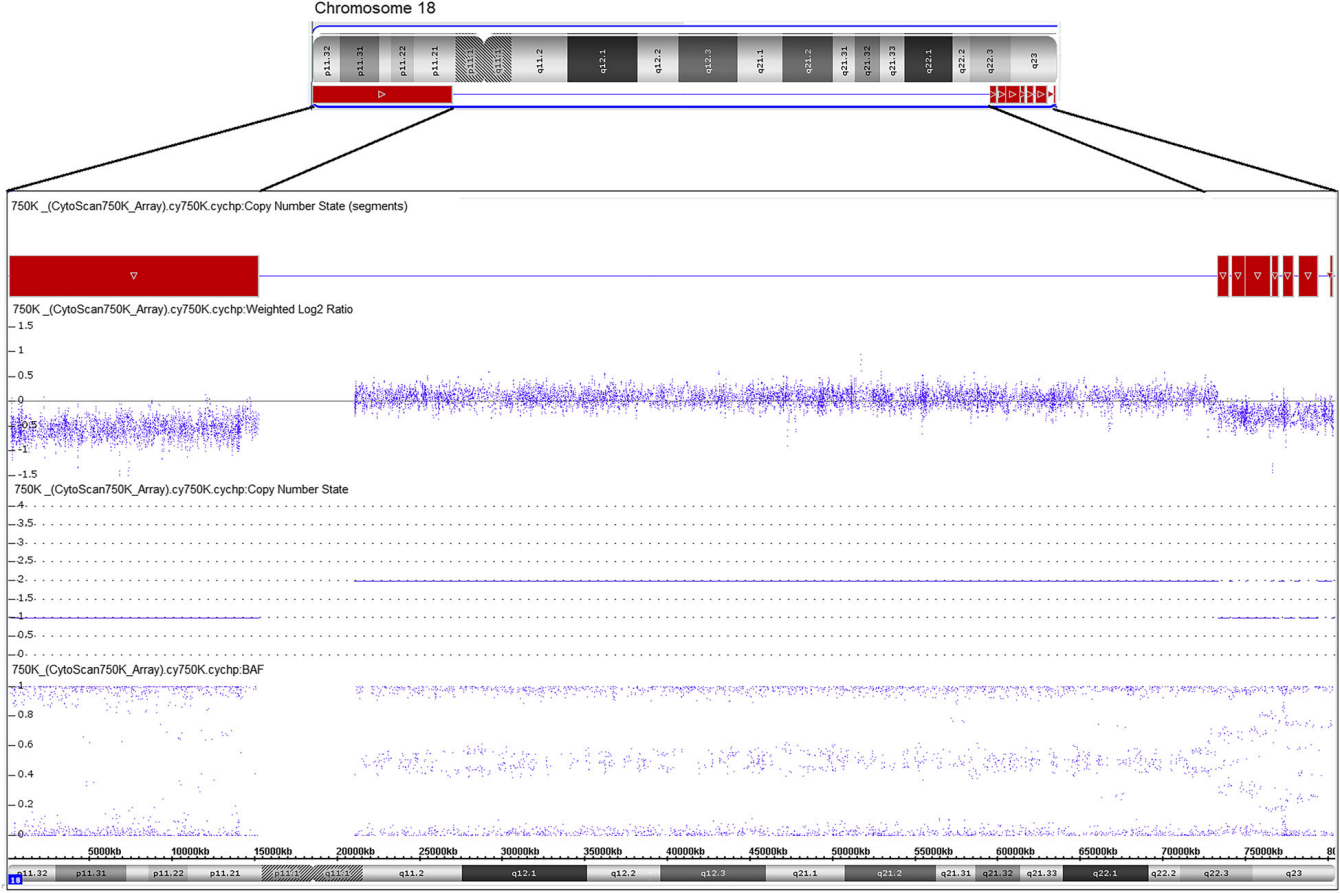
Chromosomal microarray showed 15 Mb deletion at 18p11.32p11.21 and a 6.1 Mb deletion at 18q22.3q23 i.e. arr[GRCh38] 18p11.32p11.21(136226_15181209)×1,18q22.3q23(73278051_80,255,845)×1. The figure shows, predicted deletion segments by CytoScan software, weighted log2 ratio, copy number state estimated by CytoScan software and B allele frequency

Further molecular cytogenetic studies were carried out to in order to clarify the structural rearrangements. Assessment of breakpoints on chromosome 18 was carried out using Affymetrix CytoScan™ 750 K SNP array on peripheral blood derived genomic DNA. Data was analyzed using Chromosome Analysis Suite (ChAS) that revealed multiple CNV calls on #18: arr [GRCh38] 18p11.32p11.21(136226_15181209)× 1,18q11.2q21.2(24160227_51024296)×2~3,18q22.2q23(69,349,902-80,255,845)×1 ~ 2,18q22.3q23(73,278,051_79,324,294)×1,18q23(80,132,201_80,255,845)×1. This identified a partial terminal 15 Mb deletion at chromosome 18 from 18p11.32p11.21, which was previously undetected by karyotyping (Fig. 2). Additionally, multiple CNVs showing mosaic gain and loss along with terminal deletion on q- arm were observed. Our hypothesis was that these may be due to various structural rearrangements observed on chr18 via karyotyping.

**Fig. 3:**
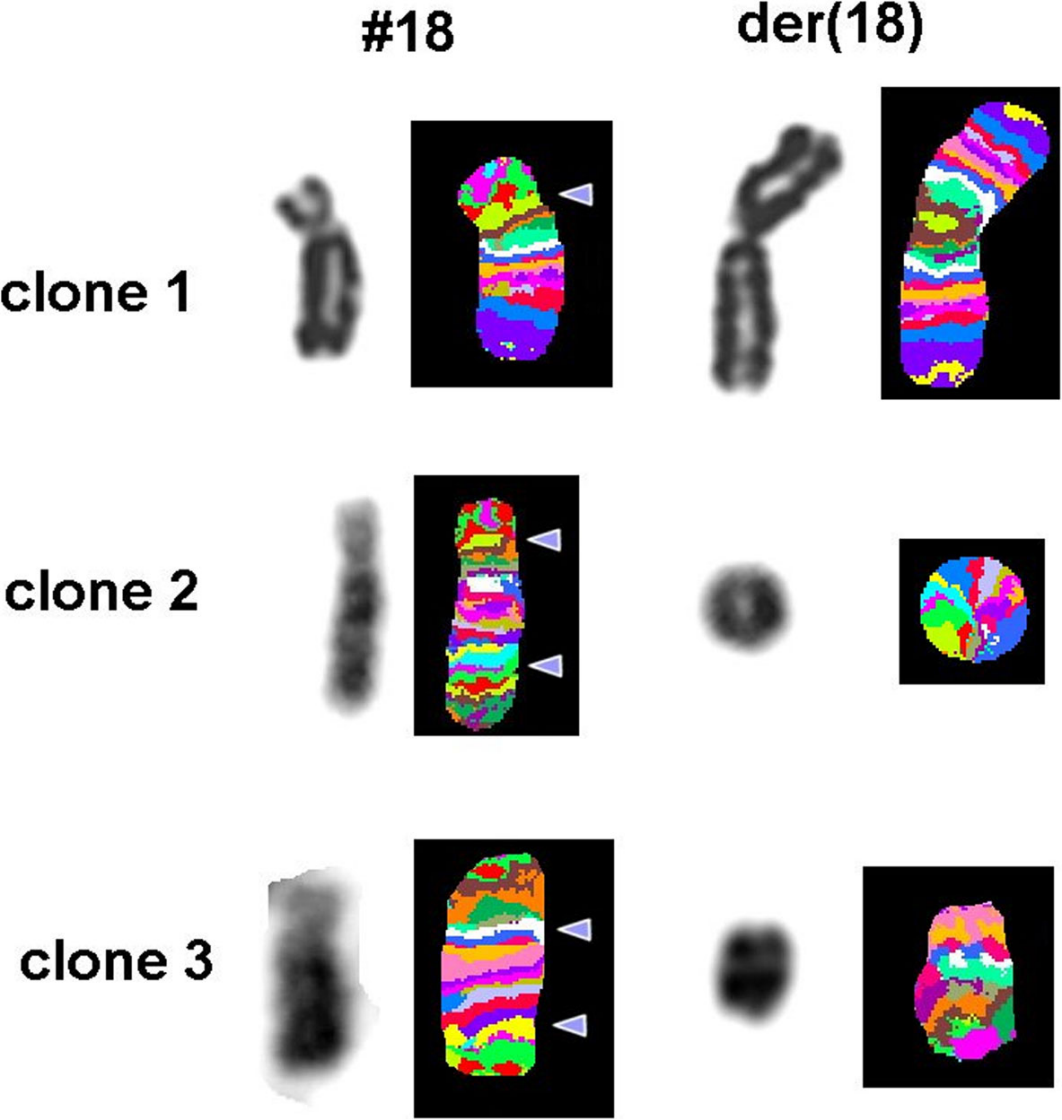
Multicolour banding (MCB) FISH study showed clone 1 containing pseudo isodicentric chromosome of chromosome 18p11.21 [psu idic (18)(qter➔p11.21::p11.21➔qter)], clone 2 containing ring chromosome 18 [r (18)(::p11.21➔q22.2::)], and clone 3 containing intertsitial deletion of 18q [del (18)(q11.2q22)] i.e. mos 46,XX,der (18)(qter➔p11.21::p11.21➔qter) [13]/46,XX,r(18)(::p11.21➔q22.2::)[12]/46,XX,del(18)(q11.2q22)[3]. Due to break and fusion in derivative del(18)(q11.2q22) pseudocolours change and the underlaying alteration can only be followed up correctly in fluorochrome profiles (result not shown)

**Fig. 4:**
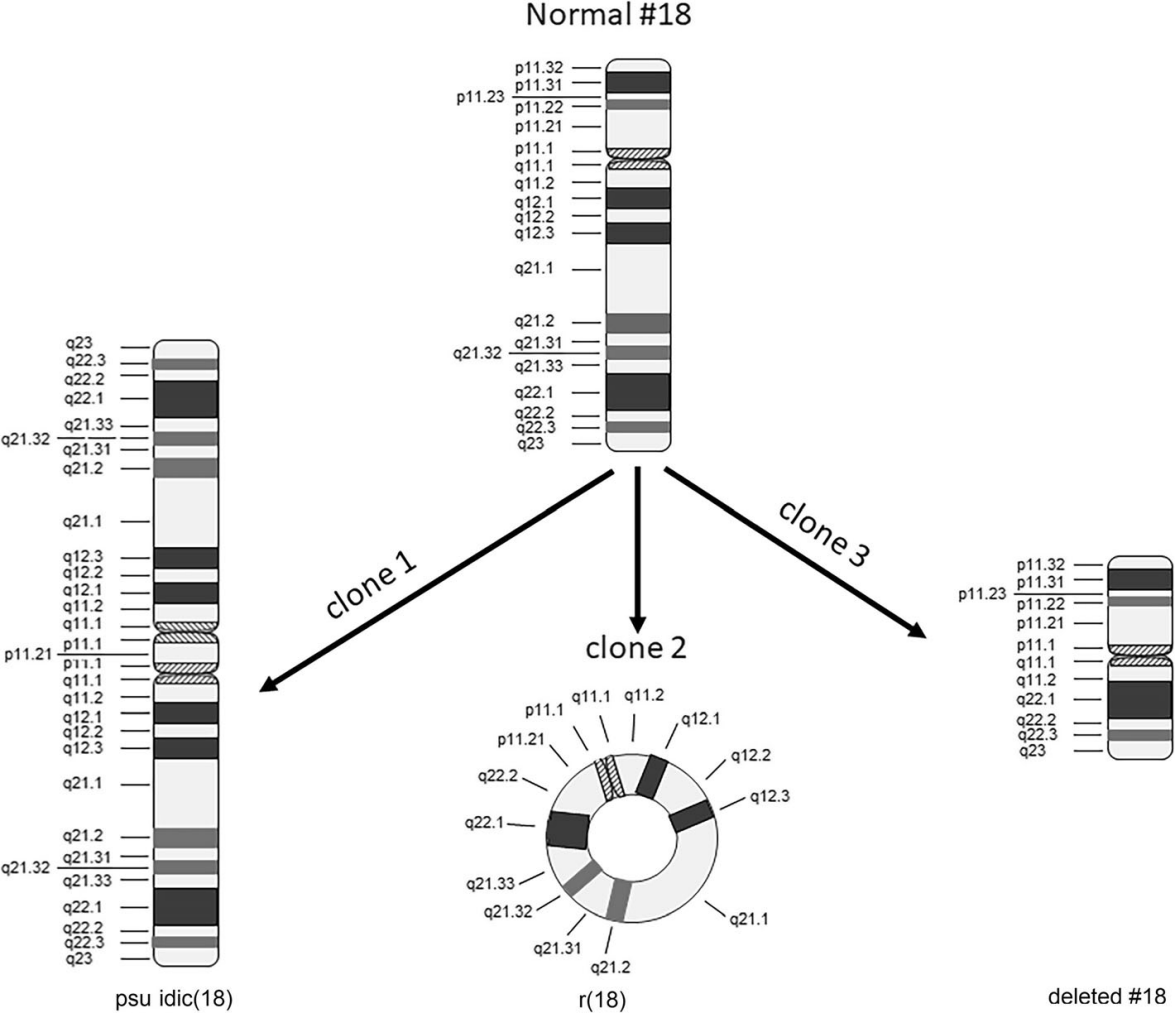
Diagrammatic representation of mosaic chromosomal abberations observed in the patient. Clone 1 consists of a pseudo isodicentric 18, clone 2 consists of a ring chromosome and clone 3 consits of deleted chromosome 18

However, to further characterize these complex structural anomalies and assess the distribution of different cell lines, we carried out multi-color banding (MCB). We utilized MCB probes encompassing whole chromosome 18, as described previously [13], and twenty-eight metaphases were evaluated. MCB enabled detection of a third cell line in addition to the two cell lines identified by the G-banded karyotype (pseudo isodicentric chr18 and r18) and further resolved the chromosome architecture as mos 46,XX,der (18)(qter➔p11.21::p11.21➔qter)[13]/46,XX,r (18)(::p11.21➔q22.2::)[12]/ 46,XX,del(18)(q11.2q22)[3] (Figs. 3 and 4). The del(18)(q11.2q22) can also be described as der (18)(pter➔q11.2::q22➔qter). No further evidence of rearrangement was seen with this study.

## Discussion and conclusions

The severity of the phenotypic manifestation depends on the nature and extent of the chromosomal abnormality and degree of mosaicism. In addition, the proportion of cell lines with different structural chromosomal rearrangements is also critical in the expression of a given phenotype (Table 1) [10]. In the present case, partial deletion of chr18p (p11.2➔pter; pure monosomy) was detected in two of the three cell-lines. One of the most frequent autosomal deletions is the monosomy of short arm of chr18. Growth failure, intellectual disability of varying degree, hypertelorism and nystagmus, short nose with anteverted nostrils, wide mouth, prominent upper lip, micrognathia, and large, protruding, low-set ears, depressed nasal bridge, short neck, wide mouth and widely spaced nipples are features usually seen in the 18p deletion, as seen in our case as well [14, 15]. Rarer or only occasionally observed malformations include premature craniosynostosis, as also seen in our case [16]. The mean age at diagnosis for craniosynostosis (oxycephaly in our case) is around 6 years. Elevated intracranial pressure is also a common clinical feature (affecting more than 60% of the patients). Many of the children have intellectual deficits and ophthalmic complications like papilledema, however, ophthalmic complications were not observed in our case [17].

**Table 1:** Comparision summary of clinical presentation in cases with isochromosome 18(q) and r (18)

Clinical features	Souraty et al. 2009	Madan et al. 1981	Bocian et al. 1993	Present case
**Age at diagnosis**	2.5 years	NA	18 months	7 years
**Sex**	Girl	Girl	Girl	Girl
**IUGR**	+	NA	Normal	Normal
**Dysmorphism**	+	NA	+	+
**Short stature**	+	NA	NA	+
**Major Abnormalities**	Cleft/lip palate, umbilical hernia, thoracic hemi-vertebrae, short claviculae, Ventricular Septal Defect	Midline cleft lip, hypotelorism, absent olfactory nerves, fused frontal lobes with mono ventricles	Asymmetrical face, short left leg	Asymmetry of face, pectus excavatum, scoliosis
**Microcephaly**	+	+	+	+
**DD/ID**	+	+	+	+
**MRI**	Cortical atrophy, enlarged ventricles, thin corpus callosum	NA	Normal	Craniostenosis
**ECG findings**	Ventricular Septal defect	NA	Normal	Normal
**Skin pigmentation**	Normal	Normal	Hypomelanosis of Ito	Hypo/hyper pigmentation on skin
**Other cognitive and behavioral problems**	Language impairment	NA	NA	Language impairment
**Karyotype**	mos 46,XX,i(18q). ish i(18q)(RP11- 417H13-,RP11-151D11-,2Xba+,RP11-10G8+/+,RP11-627G18+/+,RP5-964 M9+/+)[92]/46, XX,r(18q).ish r(18)(RP11-417H13-,RP11-151D11-,2Xba+/+,RP11-10G8+/+,RP11-627G18 +/+, RP5-964 M9-)[8]	mos psu idic(18)(p11)[64]/r(18)[36]	mos 46,XX,del(18)(p11.23)[89]/46,XX,idic(18)(p11.23) [7]/46,XX,r(18)[4]	mos 46,XX,der(18)(qter➔p11.21::p11.21➔qter) [13]/46,XX,r(18)(::p11.21➔q22.2::) [12]/46,XX,del(18)(q11.2q22) [3]

*NA* Not available, *IUGR* Intra uterine growth retardation, *MRI* Magnetic resonance imaging, *ECG* Electrocardiogram, *DD* Developmental disability, *ID* Intellectual disability, + = present

The association between holoprosencephaly and deletion of chr18p was first described by Johnson and Bachman 1976 [18] and 18p11.3 is defined as one of the critical regions for holoprosencephaly (HPE) [19]. The case reported here showed deletion on 18p11.2 however HPE was not observed. Abnormal skin pigmentation as a phenotypic feature in chromosomal mosaicism involving chr18 has been previously reported confirming hypomelanosis of Ito [10], however, our current case portrayed only mosaic skin pigmentation as previously reported [20, 21]. Neurocutaneous syndromes have nonspecific neuroradiologic abnormalities with variable frequency, like hypoplasia of corpus callosum, periventricular white matter damages, cerebral atrophy, porencephalic cysts, neuronal heterotopias etc., which this child didn’t have [22].

Three different structural rearrangements were seen in the present case. The major two cell lines- pseudo isodicentric chr18 (qter➔p11.21::p11.21➔qter) and r18 (::p11.21➔q22.2::) were seen almost with same proportion. Pseudo isodicentric chr18 was confirmed by both karyotyping and MCB in our case. We also demonstrated that the isochromosome was dicentric with two genetically identical arms which is observed rarely as compared to monocentric isochromosome. Formation of dicentric isochromosomes demands breakage and subsequent U-type reunion of sister chromatids conjoined with a partial loss of p arm. Such an event can be hypothesized to occur as a part of breakage of a chromatid followed by failed or improper DNA repair. The current patient demonstrated an isochromosome along with two alike chromatids. Therefore, we propose that the event could have happened during the second meiotic division; with no recombination occurring during the first meiotic division. Such a mechanism has been described previously [23]. In the proband r18 was confirmed solely using MCB-FISH. Its formation might have occurred simultaneously as an independent event with second break on the q arm leading to the formation of ring chromosome. Thus, two independent events causing three strand breaks might have resulted in to isodicentric chromosome along with the ring as suggested by Madan et al. [9]. Coexistence of r18, partial 18p and 18q deletion further strengthens the proposed mechanistic hypothesis. In addition, absence of cell line with normal chr18 indicate that the rearrangements occurred before conception in the germ cells. Only two cases have been published so far with a multiple rearrangement to the best of our knowledge [9, 11].

The correlation of phenotype with genotype in ring chromosome carriers is difficult to conduct due to the size of deletion and the number of genes involved at each end of the chromosome. Therefore, it becomes imperative to determine primary deletions and secondary loss/gain associated with ring chromosome formation that may occur due to the instability of the ring as observed in the present case. The occurrence of r18 together with a duplication of a chr18 segment in acentric form is occasional [2, 9, 11]. Small percentage of the cells observed with interstitial deletion chr18 was comprehensively characterized solely by MCB technique though the fragment looked bigger than r18 under microscope (Fig. 3).

In conclusion, the patient reported herein presents a novel set of chromosomal abnormalities that have never been reported before to occur concurrently. Mosaicism of two or more cell lines with unbalanced structural aberrations is an extremely rare phenomenon. However, it involves chr18 in majority of the cases [24]. The combinatorial use of molecular cytogenetics modalities invariably serves a great value in the comprehensive characterization of the structurally altered chromosomes. In addition, it helps to elucidate genotype-phenotype correlation facilitating the search for candidate genes.

## Data Availability

Data generated and analysed from the Affymetrix CytoScan™ 750 K SNP chip during this study is available as Supplementary File 1 in this published article.

## Abbreviations

CDH7: Cadherin 7
CMA: Chromosomal microarray
CNV: Copy Number Variation
CTDP1: Carboxy-Terminal domain phosphatase 1
FISH: Fluorescence in situ hybridization
MCB: Multicolor banding
ISCN: International System for Human Cytogenomic Nomenclature
r(18): Ring chromosome 18
SNP: Single nucleotide polymorphism
HPE: Holoprosencephaly
